# A Novel Method for Digital Pain Assessment Using Abstract Animations: Human-Centered Design Approach

**DOI:** 10.2196/27689

**Published:** 2022-01-07

**Authors:** Nema Rao, Sophy Perdomo, Charles Jonassaint

**Affiliations:** 1 Microsoft Inc SharePoint Spaces Seattle, WA United States; 2 Center for Behavioral Health and Smart Technology Department of Medicine University of Pittsburgh Pittsburgh, PA United States

**Keywords:** pain, pain measurement, chronic pain, animations, mobile apps, human-centered design

## Abstract

**Background:**

Patients with chronic pain face several challenges in using clinical tools to help them monitor, understand, and make meaningful decisions about their pain conditions. Our group previously presented data on Painimation, a novel electronic tool for communicating and assessing pain.

**Objective:**

This paper describes the human-centered design and development approach (inspiration, ideation, and implementation) that led to the creation of Painimation.

**Methods:**

We planned an iterative and cyclical development process that included stakeholder engagement and feedback from users. Stakeholders included patients with acute and chronic pain, health care providers, and design students. Target users were adults with acute or chronic pain who needed clinical assessment and tracking of the course of their pain over time. Phase I (inspiration) consisted of empathizing with users, understanding how patients experience pain, and identifying the barriers to accurately expressing and assessing pain. This phase involved understanding how patients communicate pain symptoms to providers, as well as defining limitations of current models of clinical pain assessment tools. In Phase II (ideate) we conceptualized and evaluated different approaches to expressing and assessing pain. The most promising concept was developed through an iterative process that involved end users and stakeholders. In Phase III (implementation), based on stakeholder feedback from initial designs and prototypes of abstract pain animations (painimations), we incorporated all concepts to test a minimally viable product, a fully functioning pain assessment app. We then gathered feedback through an agile development process and applied this feedback to finalizing a testable version of the app that could ultimately be used in a pain clinic.

**Results:**

Engaging intended users and stakeholders in an iterative, human-centered design process identified 5 criteria that a pain assessment tool would need to meet to be effective in the medical setting. These criteria were used as guiding design principles to generate a series of pain assessment concept ideas. This human-centered approach generated 8 highly visual painimations that were found to be acceptable and useable for communicating pain with medical providers, by both patients with general pain and patients with sickle cell disease (SCD). While these initial steps continued refinement of the tool, further data are needed. Agile development will allow us to continue to incorporate precision medicine tools that are validated in the clinical research arena.

**Conclusions:**

A multiphase, human-centered design approach successfully resulted in the development of an innovation that has potential to improve the quality of medical care, particularly for underserved populations. The use of Painimation may especially benefit the medical care of minority populations with chronic and difficult-to-treat pain, such as adults with SCD. The insights generated from this study can be applied to the development of patient-reported outcomes tools that are more patient-centered, engaging, and effective.

## Introduction

### Background

Pain is the number one reason people access the health care system. The Centers for Disease Control and Prevention (CDC) reported that in 2019, approximately 20.4% of US adults had chronic pain, and 7.4% had high-impact, chronic pain. Similar statistics have been reported in Canada (18.9%) and Australia (17.9%), whereas in the United Kingdom the numbers are much higher (35%-51.3%) [1-4]. The cost of medical treatment and lost productivity due to pain exceeds US $635 billion each year in the United States, more than the cost of treating cardiovascular disease, cancer, or diabetes [5]. Chronic pain also significantly affects an individual’s quality of life, negatively impacting their ability to engage in day-to-day activities, and increasing risk for depression, anxiety, and opioid dependence [6,7].

Despite the significant impact of pain on population health outcomes, pain remains inadequately assessed in the health care setting [8]. Pain is a complex sensory and emotional experience that is often difficult to communicate [9]. Unidimensional pain measures, such as the numeric or visual analog pain scale, reduce the complex, multifaceted nature of the pain experience to a single number between 0 and 10 [10]. This oversimplification not only results in poor assessment of potential physiological mechanisms but also ignores the complex roles the patient’s thoughts and mood play in the patient’s pain experience [8].

In some subspecialty medical clinics, multidimensional measures are used, such as the McGill Pain Questionnaire [11], that attempt to take into account other facets of pain beyond intensity, such as pain location, quality, and affective response. However, these measures are often overly complex and rely on long lists of adjectives or esoteric phrases to describe pain that may alienate individuals with low literacy, individuals with dementia or other cognitive limitations, non-native English speakers, and many others with communication limitations [12,13]. With the current state of clinical pain assessment, even individuals without language limitations can have their needs misinterpreted, their symptoms ignored, or their credibility challenged [14]. Ineffective communication about pain may result in patient–clinician discordance, leading clinicians to intervene on poorly described and ill-defined targets, and patients to feel misunderstood and lose trust in their provider [15,16]. The inadequacy of pain assessment tools compromises medical providers’ ability to deliver quality care and improve clinical outcomes for their patients [10,17,18].

### Painimation

To address the limitations of standard pain assessment, we used human-centered design methods to discover, design, and develop a novel method for assessing pain that leverages digital animations that we call *painimations* [19]. In this work, we hypothesized that an animation-based pain assessment tool would be more acceptable to patients with pain than traditional numerical and adjective-based pain assessments. Our work is particularly timely, given the recent promising evidence suggesting that digital health interventions are feasible, acceptable, and efficacious in a range of chronic medical conditions [20-25].

Our prior publication presented data comparing participants’ selection of painimations with their scores on validated, traditional pain scales that rely on pain adjectives and numerical scales [17]. This paper describes our process of using human-centered design to understand how patients experience and express their pain, how clinicians assess and diagnose pain, and how leveraging these observations led to the creation of a novel method for pain assessment: *Painimation*.

Our approach incorporated human-centered design principles, qualitative methods, and stakeholder engagement, and consisted of 3 distinct phases: the inspiration phase, the ideation phase, and the implementation phase [26,27]. After detailing the discovery and development process for a novel, animation-based pain assessment approach, we present initial user testing of the painimations, or abstract animations that can be visually configured to reflect pain quality, pattern, and intensity, as well as the overall Painimation prototype. Finally, we describe future directions for the use of Painimation and discuss how this digital animation approach has the potential to significantly improve medical assessment and treatment of acute and chronic pain.

## Methods

### Setting

The human-centered design process that resulted in the development of a Painimation prototype took place from January 2015 to May 2016. Key stakeholders were recruited from the Pittsburgh, Pennsylvania, metropolitan area and included patients with acute and chronic pain, clinicians, clinical researchers, and design students. All participants were 18 years of age or older. This project was approved by the University of Pittsburgh’s and Carnegie Mellon University’s Institutional Review Boards.

### Phase I: Inspiration (Empathize, Understand, and Define)

#### Overview

Human-centered design is inherently an empathic process that attempts to set aside the investigators’ or designers’ assumptions about the world and gain insight into their users’ lived experience, perspectives, pain points, and needs [26]. The goal of Phase I was to *empathize* with the target user and *understand* how pain is experienced and communicated. The next step was to *define* the most prominent barriers to effective patient–provider communication, assessment, and treatment of pain in the health care setting. To accomplish this, we conducted one-on-one, in-depth, in-person interviews with patients with acute and chronic pain, clinicians, and researchers (Table 1).

**Table 1:** Questions from interviews using directed storytelling and modified think-aloud protocol.

User and stakeholder	Clinician and clinical researcher
A *successful* experience I’ve had with a clinician around my pain assessment and management was SHORT STORY	The pain assessment protocol I follow is BRIEF OVERVIEW
I describe the pain communication between myself and my clinician as ADJECTIVE	Pain assessment is part of every interaction I have with a patient YES/NO
I summarize my clinician’s understanding of and assessment of my pain as ADJECTIVE	I use the following tools LIST/DESCRIBE
I describe my communication ability as ADJECTIVE	I document in the following way ADJECTIVE
I have been asked to rate my pain intensity on a scale like this YES/NO	(Numeric) pain scales are an effective/ineffective CHOOSE tool because REASON
The experience of using the scale was ADJECTIVE	
During that interaction, I communicated the pain intensity that I felt YES/NO	

#### User and Stakeholder Interviews

Interviews were conducted using directed storytelling [28], a design ethnography method, which allowed patients with a wide range of pain experiences to be interviewed, and yielded information about the contexts in which they had experienced pain as well as descriptions of successful and unsuccessful interactions with their clinicians.

The next part of the patient interviews consisted of a modified version of the think-aloud protocol [29], a method during which participants verbalize their thought process while doing specific tasks. The aim of this portion of the interview was to understand how patients think through 2 current pain scales: the Wong–Baker faces scale and the Numeric Rating Scale [18,30]. Additionally, patients were given a recall interview prompt to understand how they have used these scales in the past to describe their pain to medical providers.

#### Clinician and Clinical Researchers Interviews

As with the patient interviews, clinician interviews were conducted using directed storytelling to learn about their expertise and experiences in interacting with and treating patients with pain. All interviews were transcribed for later analysis.

### Phase II: Ideation (Generate Concepts and Designs)

#### Overview

The goal of Phase II was to develop solutions to the problem defined in Phase I: how to best allow patients to express their pain and facilitate pain communication with health providers.

#### Ideation and Concept Development

Analysis of the interviews from Phase I combined thematic analysis and the constant comparison method [31,32]. Codes were developed via open coding of the transcripts to determine topics and themes that emerged. Input from the designers/investigators on relevant topics was also integrated, resulting in a simultaneously inductive and deductive analysis process. Based on the topics identified in Phase I, we developed a set of criteria that needed to be met for a pain communication solution to be considered successful. These criteria served as *design principles* that guided the ideation stage where the designers generated a large number of *concepts*, or creative and innovative solutions to the pain communication problem.

Once several solutions, or concepts, are developed in an unrestricted brainstorm, all of the concepts are evaluated based on the design principles defined earlier. Any concepts that do not meet all of the design principles are discarded. The remaining concepts are ranked relative to 2 axes or factors: importance (ie, potential to impact the problem) and then difficulty (eg, cost, feasibility, scalability). Final concepts are selected based on their relative importance/difficulty and developed using generative storyboards to illustrate how the concepts might function in various scenarios. To test each concept, we conducted needs validation sessions, a design method for working with stakeholders to validate or disprove early ideas, to select a viable concept, and to transition it to the user evaluation stage.

#### Painimation Drawing Exercises

The process of developing the painimations began with the words used to describe the qualities of pain on the McGill Pain Questionnaire Short Form [11], a pain assessment method that measures pain intensity and quality using 15 descriptors of pain. Drawing exercises were conducted with a group of 16 design students from Carnegie Mellon, to develop visual depictions of the more commonly used pain adjectives. For this exercise, the design students were given a list of qualitative words that are currently used on the McGill Pain Questionnaire Short Form, such as stabbing, pounding, and shooting, and were asked to draw those words, creating a low or medium version, and a high version for each word. The selected words were those most frequently presented by patients and clinicians in Phase I exercises.

#### Painimation Development

**Figure 1:**
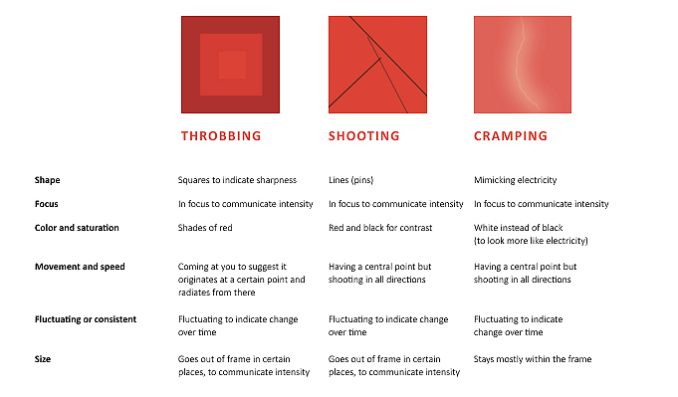
Visual considerations for painimations.

Words from the McGill Pain Questionnaire Short Form [11] were clustered into a few groups, with the idea of creating painimations that would depict and represent different sensations. The first 3 types we explored were throbbing, shooting, and cramping. Deep and dull are terms that could be applied to other qualities, so these were clustered separately. Next, the visual variables that the painimations would represent or communicate were listed. The final list included speed, saturation, focus, and size (Figure 1). Changing these variables would change the intensity of the pain depicted. These painimations were sent out to the design students in a survey with the question, “What kinds of pain do you believe these animations evoke?” The goal was to understand how participants would describe the qualities of these painimations, given the context of pain.

#### Wireframe Creation

**Figure 2:**
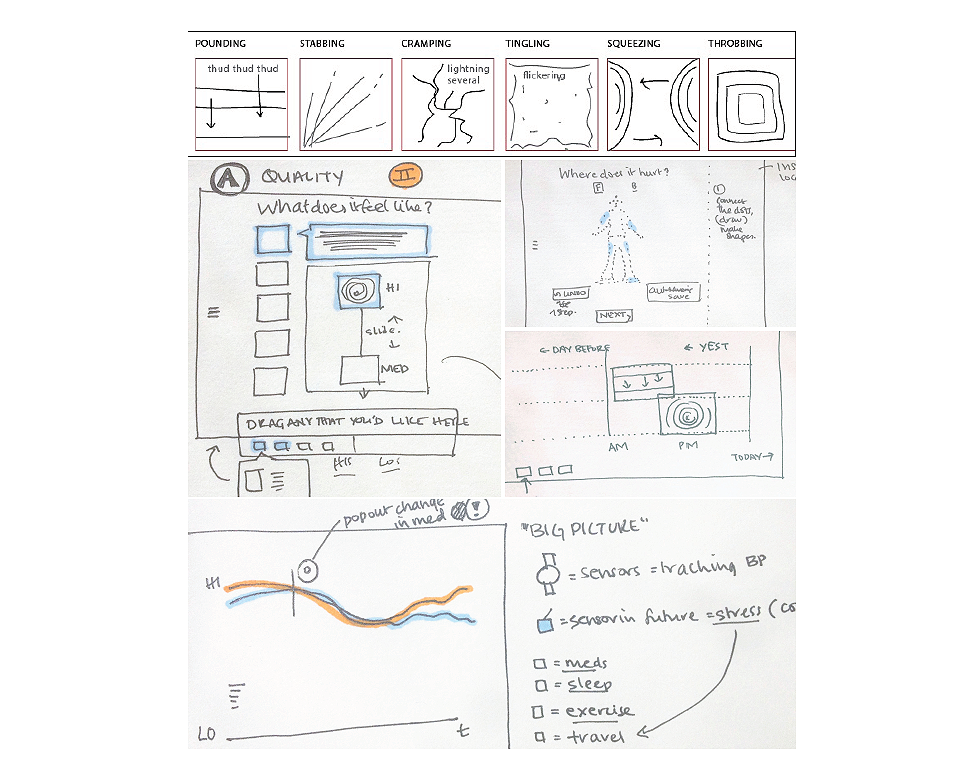
Painimation wireframe early sketches.

A new set of 11 participants—patients with a history of pain, clinicians, and researchers—were recruited by word of mouth and asked to evaluate the painimations as well as the context of use through the think-aloud protocol. Basic wireframes for the pain assessment app were created to provide context for the painimations (Figure 2). The participants were asked, “How effective do you think this tool is in aiding your pain communication?” via a modified version of the think-aloud protocol. Similarly, clinicians and clinical researchers were asked “Would something like this work? Why or why not?”

### Phase III: Implementation (Prototype, Test, and Iterate)

#### Overview

The goal of Phase III was to develop a minimally viable product to test with a small number of users. Once the painimations were refined based on user input from Phase II, we used an agile development process to build a fully functioning *prototype* of an app that utilized the painimations (ie, Painimation). A final set of 8 painimations was developed and subjected to testing and further design iteration. The designers labeled the painimations based on what pain adjective the painimations were intended to represent. Two independent, graduate-level design students were asked to identify what pain type each painimation represented. Confirmation that the painimations approximated the pain adjective they were meant to represent would allow us to transition to pilot testing; otherwise, the painimations would go through another design iteration.

#### Pilot Testing Using a Case Patient Population: Adults With Sickle Cell Disease

The use-case scenario for Painimation was the assessment and treatment of sickle cell disease (SCD) pain. SCD is a genetic blood disorder that is characterized by unpredictable vaso-occlusive episodes that lead to severe acute pain often called “crisis” and can result in long-term organ damage, chronic pain, and other complications [33]. Patients living with SCD experience pain crisis as early as infancy, and the pain can transition to chronic pain during adolescence and young adulthood. Further, SCD primarily affects underserved, racial/ethnic minorities, and patients often experience discrimination in the medical system [34]. Thus, adults with SCD have long, many times difficult, historical experience with pain and communicating pain to medical providers; these conditions informed the development of this tool.

Participating adult patients with SCD and self-reported chronic pain were presented the 8 painimations and asked, “Would you find this animation applicable to your pain?” These patients were also asked about what types of pain they experience, how they track pain, and their history of pain communication interactions with providers.

## Results

### Phase I: Inspiration (Empathize, Understand, and Define)

#### User and Stakeholder Interviews

In total, 10 patients were interviewed, 6 with acute pain (mean age 42.5 years; range 25-50; 50% [n=3] female) and 4 with chronic pain (mean age 40.0 years; range 24-58; 75% [n=3] female). Participants with acute pain had experience with temporary bouts of pain lasting no more than a few days, and patients with chronic pain had a range of pain experiences all lasting more than 3 months. Participants with acute pain experienced a hairline fracture, kidney stones, a pulmonary embolism, postsurgery pain, a root canal, and a urinary tract infection, whereas those with chronic pain experienced migraines, fibromyalgia, vulvodynia, and chronic back pain. Patients with acute and chronic pain both reported having experience communicating pain with clinicians in the medical setting.

Directed storytelling interviews revealed that patients with acute and chronic pain both felt their exact pain was impossible to communicate due to its subjective nature and the individual response to it, both physical and mental. Patients with chronic pain expressed that they particularly struggled to find clinicians who knew and accepted their conditions.

Patients described communication about pain with their health provider as “successful” if they felt heard and understood. Likewise, pain communications were described as “unsuccessful” if there was a lack of understanding, feelings of being dismissed, or intimidated. Textbox 1 displays extracted quotes from these interviews.

The think-aloud protocol revealed that patients with chronic and acute pain both expressed some confusion around traditional pain scales because they felt these scales were “vague” and “ambiguous.” For example, several patients stated they had “no clue” what “worst possible pain” in the numerical pain scale meant.

Additionally, patients felt that traditional pain scales “lack specificity” and do not accommodate detailed answers. For example, on the numerical pain scale, one might want to say, “It’s an 8 when I am applying pressure, and a 7 when I am resting, and a 10 early in the morning.” Patients said that they used these scales to communicate their pain intensity because they had to; 5/10 respondents said their numerical pain rating did not feel accurate.

**Extracted descriptors for clinical communication.**

*Successful*

- Personable, friendly
- Professional
- Dead-on
- Light at the end of a tunnel
- Calming
- Relieving
- I felt in control
- I was actually being heard
- Improved over time

*Unsuccessful*

- Zero understanding
- Accused me of lying
- Impossible
- Dismissive
- Limited
- Intimidating
- I felt stupid

#### Clinician and Clinical Researchers Interviews

A total of 7 individuals were interviewed, 4 clinicians (mean age 36.3 years; range 30-50 years, 50% [n=2] female) and 3 clinical researchers (mean age 50.7 years; range 36-58 years; 33% [n=1] female). Clinicians had experience in emergency medicine, general medicine, and physical therapy while clinical researchers had experience in clinical psychology, hematology, and anesthesiology. Clinicians had experience caring for patients with chronic and acute pain, while clinical researchers provided their clinical experience as well as a rich perspective into current research, challenges, and opportunities.

Directed storytelling interviews revealed clinicians’ and clinical researchers’ perspectives on traditional pain scales. Clinicians explained that a numeric value on the Numerical Pain Rating Scale is only meant to represent one person’s pain: “one person’s 5 can be compared to their 9, but you cannot compare two individuals’ 9’s.”

A numeric value is useful for communications between clinicians and provides a system that is well understood universally across the medical system. Numeric scales are especially useful in the context of postsurgery pain when clinicians are not as interested in the number itself as in whether the medication or treatment has been effective in reducing pain. In fact, the numeric scale was designed to provide a system for clinicians to note progression in acute and curable pain. Still, some clinician participants stated that in the emergency room, there is some aversion to the numeric system, because patients may exaggerate or falsify their pain score to receive treatment. There was a general belief from respondents that the emergency room sustains the problem of addiction because they cannot deny opioid treatment to patients who report high pain scores, especially if they have an outpatient opioid prescription.

### Phase II: Ideation (Generate Concepts and Designs)

#### Painimation Concept Development

Based on the thematic insights taken from analysis of the user and stakeholder interviews, we established a set of design principles as criteria to support the creation of concept storyboards. A successful solution to the pain communication problem would meet all criteria listed in Textbox 2.

**Design principles as criteria.**

*Aid patient in describing pain*

Given the scope of this project, the attempt was not to remove patient description or report altogether (with automated pain detection, for example) but rather to support that verbal description.

*Quantitative representation of pain*

Patients want to know that their qualitative experiences matter as much as the quantitative selection. Clinicians, by contrast, required a number of some type that can indicate pain severity and show treatment-related improvements.

*Personalized*

Patients need to feel that assessment is personalized to them and their pain thresholds. With chronic pain it is all the more important to allow conversations to address the patient’s individual journey and take into account changes in their pain experience over time or even moment to moment.

*Concise*

Because time is limited (and pain assessment is just one part of the interaction between the patient and clinician), the procedure needs to be short and simple to complete, yet provide the necessary data to guide diagnosis and treatment.

*Facilitate the conversation*

Based on the study findings, the most prominent stakeholder need was for a tool that would improve the patient–provider interaction by making the communication surrounding pain symptoms easier, and helping patients feel heard and understood. The relationship between the patient and clinician was viewed as the most important aspect of the medical encounter.

From the ideation session and concept selection process as described in the “Methods” section, 3 final concepts were selected and then developed out using generative storyboards to illustrate how the principles might fit into various scenarios. The 3 concepts were (1) expressive pain painimations, where patients would use animations to describe their pain to providers; (2) a personalized pain threshold scale, where rather than being restricted to a 0-10 scale patients would use an app to set their highest and lowest pain based on their own descriptors, words, or numbers; and (3) communication-style matching, where patients would be matched with a provider that fits their communication style. Needs validation sessions revealed that only the painimation concept qualified as both desirable and feasible for both patients and clinicians. Patients felt the painimations were more expressive than words or images alone, had an emotional quality, and even incorporated the fluctuations of pain over time. Clinicians and clinical researchers believed the painimation concept could work in their clinic and felt the concept would help create rapport between patients and clinicians.

In terms of feasibility, patients felt there might be individuals who prefer words over images or may not understand the painimations. It was evident that any tool would need to be very easy to understand. Clinicians and clinical researchers expressed that they would still need a number and method to translate the painimations into a score that can indicate severity or be used to compare with the traditional 0-10 numeric pain scale, or other pain assessment measures.

#### Painimation Drawing Exercises

**Figure 3:**
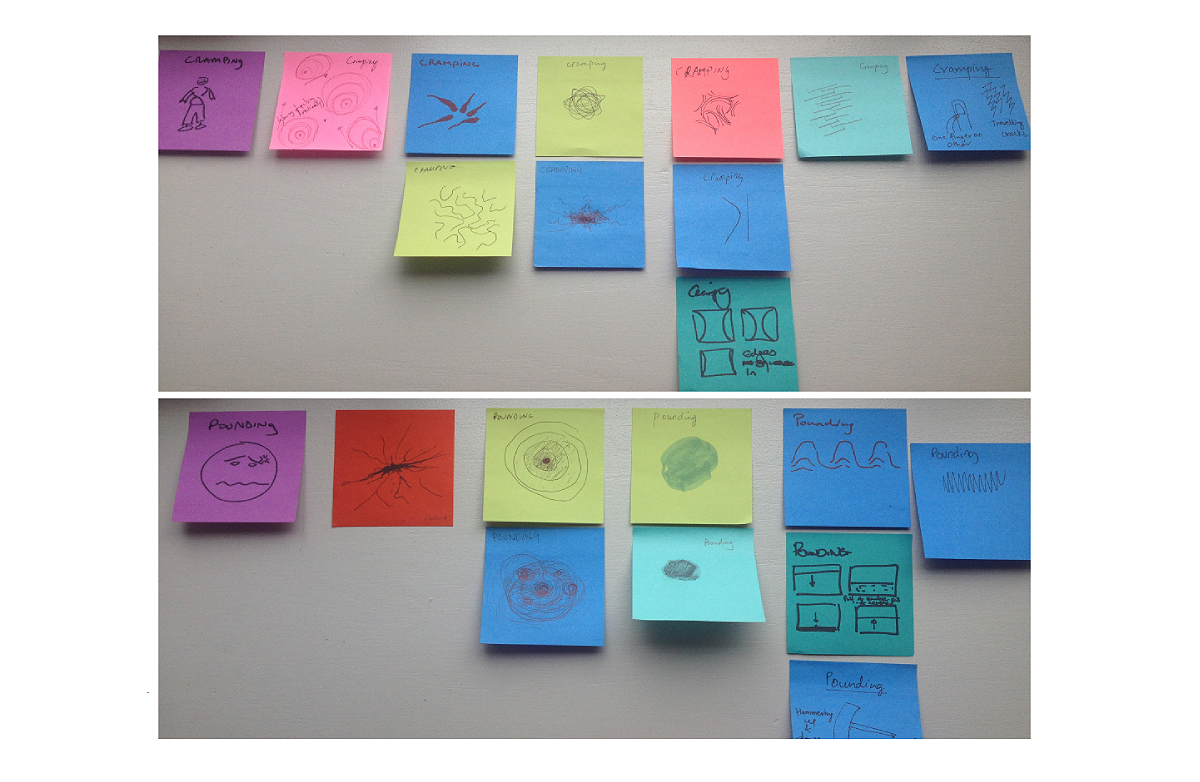
Clustering of participant drawings.

As part of the painimation development process, a total of 16 graduate design students participated in a drawing exercise where they were asked to draw a series of pain adjectives from the McGill Pain Scale. The student drawings were then clustered based on approach and also arranged according to intensity. This exercise resulted in drawings that were quite similar. The drawings were grouped by similarity, and the final groupings were used to inform the initial set of painimations (Figure 3).

First, a low-medium intensity version was created for the initial 3 painimations (throbbing, cramping, and shooting). Next, 2 new painimations (pounding and tingling) were created, each with a high and medium value. These 2 words came from the original list and were created to provide more variety in the painimations to allow for a range of responses.

#### Painimation Development

In addition to the drawing exercise session, the graduate design students participated in a survey to evaluate a preliminary set of painimations based on early findings. The “what kinds of pain do you believe these animations evoke?” survey of throbbing, cramping, and shooting painimations revealed rich language within the responses, which were organized into emergent themes: recall, time + change, and representation (Textbox 3). Participants used the painimations as a starting point to recall pain incidents and memories. They mentioned the temporal or changing nature of pain. Additionally, participants indicated satisfaction and comfort using these painimations to represent a sensation.

The throbbing painimation had the highest responses of 1 particular word, which was “throbbing” (n=11). For the shooting painimation, “quick” and “sharp” had the same number of occurrences (n=5). The cramping painimation had a tie between “dull,” “deep,” and “slow” (n=2). Because of this lack of convergence, the cramping painimation was revised. Multimedia Appendix 1 displays word frequency in responses.

**Emergent themes from survey of painimations.**

*RECALL*

Participants used the animations as a starting point to recall certain pain incidents and memories.

Example quotes:

*Reminds me of when I was having my broken arm bent by a pair of nurses to be put into a cast.*

*Like when I come in from outside when it is cold and my ears heat up uncomfortably, or if I jam my finger and it swells to the point I can feel my heartbeat in my finger.*

*TIME + CHANGE*

Participants mentioned the temporal or changing nature of pain.

Example quotes:

*Pain that fluctuates in intensity.*

*Very erratic pulsing.*

*Something that starts out in one area and spreads across the body.*

*Coming up and then dying back down.*

*Slowly beginning with mild intensity, rising in a crescendo to a near-blinding, wince-inducing pain.*

*REPRESENTATION*

Participants indicated satisfaction and comfort with using these animations to represent a sensation.

Example quotes:

*This feels like it could describe that pain well.*

*I think the strong visuals might really speak to some people.*

*This could easily resemble how I felt when I got my wisdom teeth out.*

#### Creation and Evaluation of Wireframes for a Painimation app

A new group of 5 patient participants and 4 clinician researchers was asked to interact with wireframes of an app that used painimations to measure pain. Feedback included participants wanting to see the whole set of painimations, so they knew how many choices they had. They also preferred that the intensity be depicted through a slider.

**Figure 4:**
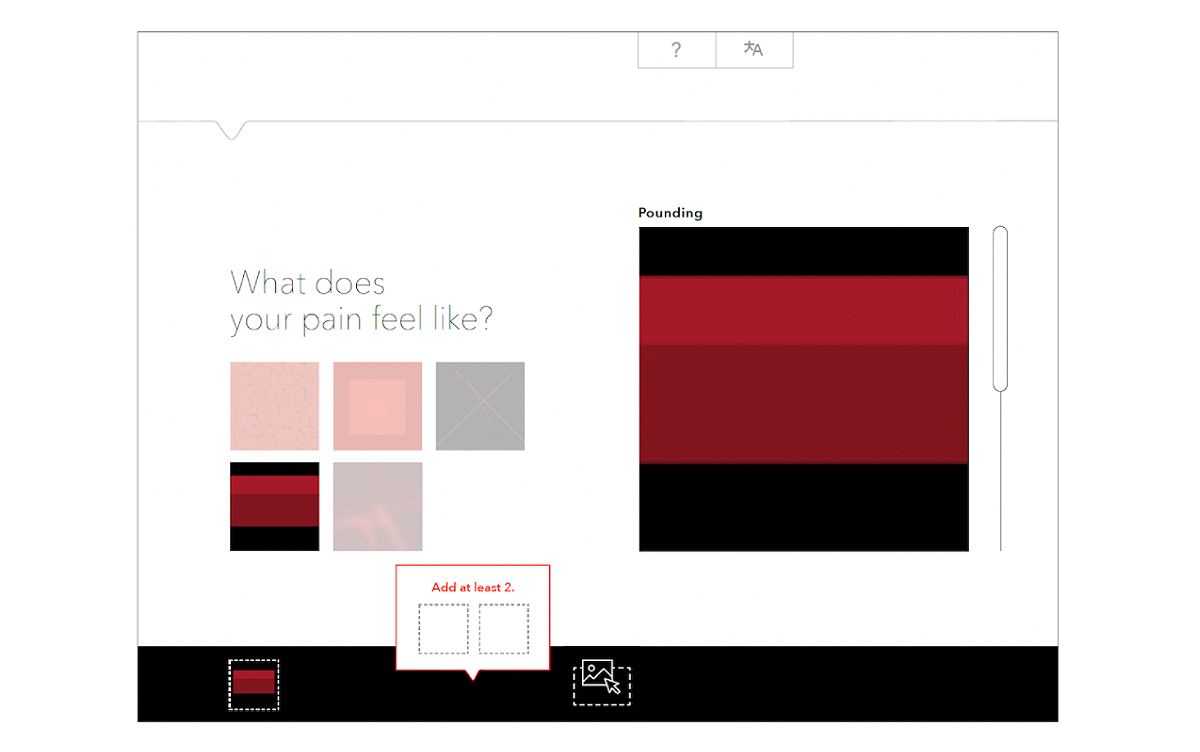
Painimation selection wireframe.

To resolve participant concerns, we created an instruction page to precede the viewing of actual images, on how to choose the painimations and increase and decrease the intensity; thumbnails of all painimations were shown on each screen with textual description, and arrows were replaced with a prominent slider. To provide users with feedback after making their selections, a panel was added at the bottom where the chosen painimations could be dragged and dropped (Figure 4).

### Phase III: Implementation (Prototype, Test, and Iterate)

#### Final Set of Painimations

Based on user feedback throughout Phase II, a final set of 8 painimations were developed and then independently reviewed by 2 graduate design students outside of the investigative team (see example 2 in Multimedia Appendix 2).

The 2 graduate students were asked to label each painimation using a provided set of pain adjectives. Their labeling of the painimations approximated the intended representations, confirming that a broad set of pain types was depicted as unique feature sets, with no overlap between them.

**Figure 5:**
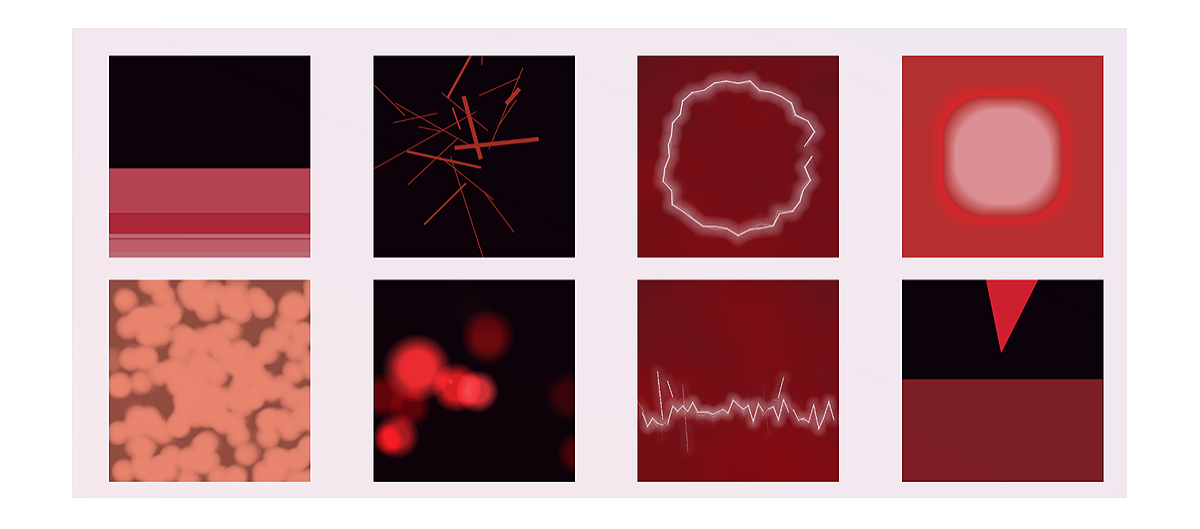
Final set of painimations.

These final 8 painimations (Figure 5) were then reviewed by the patients (n=5) and clinical researchers (n=4). Patient participants felt these painimations would aid in their pain communication, and several statements suggested that the painimations resonated. Participants would look through the set of painimations, choose 1 or 2, and make statements such as “This one really feels like my headache, exactly!” Other general comments about the idea itself included “These painimations feel like the aha moment for me. Hopefully, doctors will see it soon, too,” and “Just knowing that doctors are asking us this question with a tool that comes closer to what we’re feeling, shows that they are being empathetic and less dismissive.”

While patients acknowledged the benefits of seeing something more qualitative and contextual, they were also concerned about the limitations of the current system: “What is to stop me from getting frustrated with this system in the same way that I currently get frustrated with the number system [wanting to increase the value of the slider to more than what is possible]?”

The clinical researchers’ main concern was that the painimations needed validated numerical values of intensity. Although each painimation entry produced a numerical value of 0-100 on the slider and the painimation quality type (eg, “throbbing”), these values would need to have reliable and credible numerical correlations with the traditional numerical pain scale (eg, a particular painimation calibrated at a certain level would equal an 8/10 on the numerical pain scale).

#### Pilot Testing Painimation With Adults With Sickle Cell Disease

To confirm the acceptability and usability of the painimations, we tested a prototype of a Painimation app designed in Phase II with a use-case sample (adults with SCD-related pain). Six African American adults (age range 24-32 years, 67% [n=4] female) with SCD and self-reported chronic pain completed a pain entry using the prototype Painimation app and were asked to provide a verbal evaluation of all 8 painimations in a modified think-aloud protocol.

The adults with SCD reported that the Painimation app is more engaging, easier to use, has less entry burden, and leads to more of a conversation compared with other pain assessment forms they have used in the past.

In response to the question, “Would you find this animation applicable to your pain?” 6/6 patients with SCD responded “Yes” for electrifying; 5/6 for stabbing; 4/6 for burning; 3/6 for cramping; 2/6 for shooting; and 1/6 for throbbing, tingling, and pounding. Interestingly, 1 participant mentioned that the burning painimation looks like beginning stages of sickle cell crisis. Another patient felt that most painimations were not “severe” enough to represent her pain.

The types of pain seemed to differ between patients; however, many of the patients described their pain as stabbing and pulsating, and they consistently described some of their pain as continuous. In terms of pain tracking, 3/6 patients tracked their pain in their phone or journal, while 2 only documented pain crises, rather than daily pain. One patient said his pain did not change, so he did not feel the need to track it.

These patients echoed what patients with chronic pain in our earlier interviews reported regarding communicating with providers about pain. They liked when they felt like doctors listened and cared but were discouraged when they did not feel heard, when doctors seemed as if they did not have empathy, or did not understand their condition.

## Discussion

### Application of Painimation

Successful medical care depends on effective communication between patients and clinicians regarding the patients’ health symptoms and the most appropriate therapeutic path [35]. Providers are unable to deliver quality medical care when they lack the tools to appropriately assess or interpret patient symptoms that are critical to diagnosis and treatment. This is especially true for the assessment and treatment of pain.

Through a human-centered design approach, our study discovered that patients with pain frequently have negative interactions with providers characterized by misunderstandings, negative accusations, and intimidation. A major cause of this breakdown in the patient–provider interaction is the challenge in communicating pain and feeling understood. Patients, clinicians, and researchers in this study reported that the current pain assessment approaches used in the medical setting fail to accurately capture or communicate patients’ pain experience, have limited effectiveness for guiding diagnosis and treatment, and may exacerbate breakdowns in communications between patients and providers. Other studies have also reported that measures oversimplifying the pain experience may lead to patients’ personal legitimacy being undermined and result in clinicians inadvertently contributing to chronic pain stigmatization [36]. Given the importance of patients feeling respected and supported by their clinicians, it is imperative to improve patient–clinician communication regarding pain [37].

To address this gap, the current human-centered design study resulted in the development of a novel pain assessment approach that leverages digital animations. The use of pain animations or painimations showed promise with a use-case clinical sample of adults living with SCD-related chronic pain. Our prior published study found that patients’ selection of painimations were correlated with their scores on validated scales, and yielded some evidence that painimations may have better diagnostic potential than traditional multidimensional pain scales [17].

Given that pain is incredibly complex and its qualities are particularly difficult to express [8], there have been efforts to improve communication of pain [38-41]. For example, presenting abstract or literal pain images to patients with chronic pain during pain consultations was associated with clinician warmth and empathy, improving the patient–clinician rapport and communication [38]. Another example is Pain QuILT (a newer version of the Iconic Pain Assessment Tool), a web-based and mobile-accessible tool for the visual self-report and tracking of pain that offers 16 pain qualities, such as burning, electrical, and stabbing [39,40]. Pain QuILT was rated significantly easier to use than both the McGill Pain Questionnaire and the Brief Pain Inventory and was associated with fewer barriers to complete [40]. Our findings support and extend this work.

Abstract painimations can capture the experience of pain in a comprehensive manner. These painimations can be visually configured to reflect pain location, quality, and intensity. Moreover, they allow users to interpret the painimations instead of restricting them to specific/labeled pain quality options. The abstract and nonverbal nature of the painimations is also important because it helps level the playing field for marginalized or underserved populations. Patients with lower health literacy, communication disorders, or cultures/languages different from those of the providers have previously faced a communication gap that put them at a disadvantage when seeking medical care. While there are complicated power dynamics between a patient and a clinician, it benefits the patient to have a tool that does not rely on literacy or language, upon which to build conversation and allow patients to more effectively report their symptoms. As evidenced by this project, providing something that is removed from medical jargon or systems (which were not designed from a patient-centric perspective) allows patients to express themselves comfortably, knowing that their comments are valued, heard, and hopefully understood. Furthermore, these painimations address the disparities that current pain assessments perpetuate due to their use of complex words that may alienate individuals with low literacy, disabilities, cognitive impairment, or other communication barriers [12,13,42].

### Relevance and Importance of Human-Centered Design Work

Human-centered design and evidence-based data, together, have significant potential for disease prevention and management [43]. Patients need to have the opportunity to participate as true partners in their health care [44]. Utilizing user-centered participatory approaches allows the evaluation of which elements work best for which populations in which contexts [45]. Thus, application of human-centered design in health care will exponentially improve the effectiveness of medical care and disease prevention [43].

Human-centered design is gaining traction in health care and the proliferation of mobile technologies expands opportunities for innovation, particularly because of the wide access to smartphones in clinical populations [23,46-48]. Mobile technologies have been shown to be beneficial in reducing pain severity and are well liked by patients and clinicians [49]. In fact, a study of perspectives of patients with chronic pain on methods of assessing pain found that 80% favored use of a digital version of body template/diagram, and 43% favored use of technology [50]. However, most mobile pain technologies (around 70%) still do not systematically engage patients with chronic pain as end users during app development, nor do they involve clinicians [51]. To ensure short- and long-term engagement of mobile app or digital health interventions, it is critical to include patients and clinicians in all stages, particularly the development stages [48,52-54].

### Strengths and Limitations

This study has several strengths, including a rigorous human-centered design approach that involves target users and stakeholders at each phase. A major limitation of this study approach, however, is the small sample and thus limited age, genders, ethnicity/race, and number of pain conditions that were represented by the user and stakeholder groups. For example, only a small number of African Americans with SCD tested the app. Consequently, the generalizability of our findings is limited. The Painimation concept will need to be tested by a larger, more representative sample in terms of age, gender, and ethnicity/race with a broad range of pain types, to determine if all pain experiences are represented in the current set of 8 painimations or if additional painimations need to be designed.

Finally, the reflexivity of the investigators and consultants needs to be considered and was systematically evaluated. It is likely that prior experiences and biases may have influenced the direction of designs and how the findings were interpreted. Future work in this area will benefit from more objective evaluations of the tool and the results.

### Future Directions

This study demonstrates the process of human-centered design to build empathy for the end user and ultimately develop and implement an innovative solution for a prominent problem in medical care. Further research is needed to establish whether developing animations that explicitly measure affect and emotion would be beneficial. Additionally, how particular pain characteristics (conditions) might influence the further development of alternative methods (including this one) needs to be considered.

While these painimations have proven to have resonance with participants in this study, there is potential with augmented and virtual reality to develop the pain assessment experience further. For example, a doctoral project at the Norwegian University of Science and Technology in Trondheim is exploring how virtual reality can help nurses develop and sustain their empathy, as clinicians may become desensitized. It simulates morning sickness (nausea and dizziness, for example) through a headset that nurses wear. In relation to this project, the use of painimations in the virtual reality space, recreating the nausea or disorientation that patients with pain would experience, could lead to an intervention that would increase the empathy of family members, friends, and providers towards pain patients.

Finally, with the current data, it is unclear whether Painimation is a tool to replace other measures or to be used in conjunction with other forms of pain assessment. Further, in clinical medicine the 0-10 scale is well-established as the status quo, and health professionals will need to be convinced that using painimations offers useful and relevant information that can improve their clinical practice. Changing the pain assessment landscape is challenging and there are significant barriers to implementing new tools into routine clinical care. The current body of studies does not address how the pain conversation can be changed in this radical new direction; however, this is a starting point with potential to encourage and inspire other pain researchers to explore novel methods for assessing pain.

### Conclusions

This study provides evidence that employing a human-centered design approach in clinical research has the potential to change how medical care is practiced. Currently, most electronic patient-reported outcomes measures for pain are essentially digital copies of paper–pencil questionnaires. Computer adaptive testing has helped streamline assessments, but the fundamental method of assessing symptoms and outcomes with words and numerical scales has not advanced along with the digital era. There is a need for more human-centered design studies to explore how technology can be leveraged to radically improve and advance how patient-reported pain outcomes are assessed.

## Appendix

Multimedia Appendix 1 Word frequency in responses from the Painimation survey.

Multimedia Appendix 2 Example of two painimations.

## Abbreviations

CDC: Centers for Disease Control and Prevention
SCD: sickle cell disease
