# Supplementary material for: A Novel Method for Digital Pain Assessment Using Abstract Animations: Human-Centered Design Approach
Source: JMIR Hum Factors. 2022 Jan 7;9(1):e27689. doi: 10.2196/27689 (PMC8783278; doi:10.2196/27689)
Supplement: Multimedia Appendix 1 [file humanfactors_v9i1e27689_app1.docx]

| 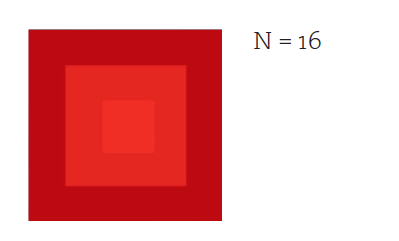 | | | | | Throbbing | | | | |  | | |
| --- | --- | --- | --- | --- | --- | --- | --- | --- | --- | --- | --- | --- |
| Adjectives | | | | | Nouns | | | | | Adverbs | | |
| Throbbing | 11 | Pulsing | 5 | Pain | | 13 | Headache | 5 | Badly | | 1 |  |
| Acute | 2 | Burning | 2 | Finger | | 2 | Ankle | 1 | Mild | | 1 |  |
| Annoying | 1 | Beating | 1 | Anxiety | | 1 | Breath | 1 | Really | | 1 |  |
| Bright | 1 | Cutting | 1 | Bruise | | 1 | Cold | 1 | Very | | 1 |  |
| Dull | 1 | Erratic | 1 | Color | | 1 | Ears | 1 |  | |  |  |
| Fluctuating | 1 | Heavy | 1 | Eyes | | 1 | Heartbeat | 1 |  | |  |  |
| Jammed | 1 | Jumping | 1 | Heat | | 1 | Hurt | 1 |  | |  |  |
| Rhythmic | 1 | Severe | 1 | Mind | | 1 | Panic | 1 |  | |  |  |
| Sharp | 1 | Swelling | 1 | Red | | 1 | Teeth | 1 |  | |  |  |
| Thumping | 1 | Twisting | 1 | Temple | | 1 | Toe | 1 |  | |  |  |
| Uncomfortable | 1 |  |  |  | |  |  |  |  | |  |  |
| 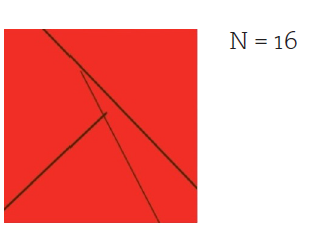 | | | | | Shooting | | | | |  | | |
| Adjectives | | | | | Nouns | | | | | Adverbs | | |
| Quick | 5 | Sharp | 5 | Pain | | 11 | Headache | 2 | NA^a^ | |  |  |
| Cutting | 3 | Shooting | 3 | Needles | | 2 | Ant | 1 |  | |  |  |
| Stinging | 3 | Intense | 2 | Bee | | 1 | Bites | 1 |  | |  |  |
| Piercing | 2 | Pinching | 2 | Bolts | | 1 | Heel | 1 |  | |  |  |
| Stabbing | 2 | Burning | 1 | Injury | | 1 | Lemon Juice | 1 |  | |  |  |
| Bursting | 1 | Darting | 1 | Light | | 1 | Migraine | 1 |  | |  |  |
| Flowing | 1 | Frequent | 1 | Nausea | | 1 | Pins | 1 |  | |  |  |
| Long | 1 | Modulating | 1 |  | |  |  |  |  | |  |  |
| Pricking | 1 | Sensitive | 1 |  | |  |  |  |  | |  |  |
| Severe | 1 | Scratching | 1 |  | |  |  |  |  | |  |  |
| Slicing | 1 | Small | 1 |  | |  |  |  |  | |  |  |
| Static | 1 | Sudden | 1 |  | |  |  |  |  | |  |  |
| Surge | 1 | Vicious | 1 |  | |  |  |  |  | |  |  |
| Throbbing | 1 |  |  |  | |  |  |  |  | |  |  |
| 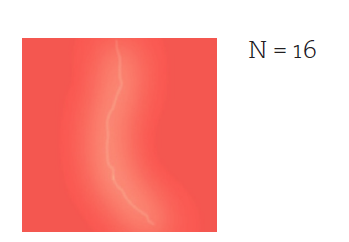 | | | | | Cramping | | | | |  | | |
| Adjectives | | | | | Nouns | | | | | Adverbs | | |
| Dull | 2 | Deep | 2 | Pain | | 17 | Headache | 4 | NA | |  |  |
| Slow | 2 | Acute | 1 | Back | | 3 | Elbow | 3 |  | |  |  |
| Aching | 1 | Bending | 1 | Muscle | | 3 | Arm | 1 |  | |  |  |
| Blinding | 2 | Broken | 1 | Body | | 1 | Cast | 1 |  | |  |  |
| Burning | 1 | Constant | 1 | Crescendo | | 1 | Force | 1 |  | |  |  |
| Continuous | 1 | Dying | 1 | Nurses | | 1 | Storm | 1 |  | |  |  |
| Exacerbated | 1 | Falling | 1 | Tissue | | 1 | Toothpaste | 1 |  | |  |  |
| Flowing | 1 | Hot | 1 |  | |  |  |  |  | |  |  |
| Hurts | 1 | Irritating | 1 |  | |  |  |  |  | |  |  |
| Mild | 1 | Muscular | 1 |  | |  |  |  |  | |  |  |
| Pressure | 1 | Pulsating | 1 |  | |  |  |  |  | |  |  |
| Shooting | 1 | Split | 1 |  | |  |  |  |  | |  |  |
| Strong | 1 | Subtle | 1 |  | |  |  |  |  | |  |  |
| Sharp | 1 | Tingling | 1 |  | |  |  |  |  | |  |  |

^a^NA: not applicable.
